# The effectiveness of clinical frailty assessment in older patients with Traumatic brain injury in predicting outcome and quality of life: a systematic review

**DOI:** 10.1007/s10143-025-03659-y

**Published:** 2025-06-16

**Authors:** Elizabeth Cray, Nida Javed Hayat, Chi Ho Song, Max Western, Ellie Edlmann

**Affiliations:** 1 Department of Neurosurgery, Southwest Neurosurgery Centre, University Hospital Plymouth, Plymouth, PL6 8DH UK; 2 Department for Health, University of Bath, Bath, BA2 7AY UK; 3 Peninsula Medical School, Faculty of Health, University of Plymouth, Plymouth, PL6 8BX UK

**Keywords:** Frailty, Ageing, Traumatic brain injury, Outcome

## Abstract

Ground-level falls are the most common cause of Traumatic brain injury (TBI) leading to emergency hospital admissions, in adults aged 65 years and older. The world’s population is ageing and frailty is becoming more relevant in healthcare provision, therefore assessment of frailty on admission is integral to care planning. Identifying whether frailty is a risk factor for poor outcomes may facilitate clinical decision making and direct care appropriately. This review aimed to evaluate the effectiveness of a clinical frailty assessment or scale in predicting outcomes in older patients following a TBI, including mortality, functional recovery, hospital length of stay, and discharge disposition. A systematic review of OVID, EBSCO, Elsevier and Wiley from 2005 to 2025. Included a majority of patients aged 65 years and over, diagnosed with a TBI with a validated frailty assessment tool and at least one outcome measure reported. A total of 12 observational studies (464,606 patients) were included, with a mean age ranging between 70 and 83 years. These studies utilised seven distinct frailty assessment tools. Falls from standing were the most common mechanism of injury identified. Frailty was associated with 30-day and 1-year mortality and unfavourable outcome in combination with a reduced Glasgow Coma Score (GCS) on admission. Frailty was an independent predictor of length of hospital stay, discharge disposition and functional recovery but no study used a validated quality of life tool. Various frailty assessment tools demonstrate effectiveness in predicting clinical outcomes when used in combination with patients age, co-morbidity and neurological evaluation. The predictive value of these tools supports their clinical utility in clinical decision making. Further prospective research is needed to understand how frailty relates to longer term outcomes, particularly quality of life, which was not measured in the included studies.

**Clinical trial number** Not applicable.

## Introduction

Traumatic Brain Injury (TBI) is a significant health concern in the elderly population. The prevalence of TBI among older adults is increasing due to an aging global population. Falls are the most common cause of TBI in older adults, accounting for 81% of TBIs in those aged 65 and older and requiring hospital admission [1, 2]. The increased risk of falls among the elderly are due to factors like impaired balance, medication side effects, and vision problems which contribute significantly to these high rates.

In the United Kingdom, it is estimated that the rate of TBI-related hospital admissions among older adults aged 65 years and over, ranges between 300 and 500 per 100,000 individuals annually [3, 4]. This rate significantly increases in adults aged 75 and over, where it can exceed 600 per 100,000 annually [3, 4]. Older adults who have sustained a TBI are associated with worse functional outcomes when compared to younger adults, regardless of injury severity [5, 6]. Mortality following a severe TBI increases with older age, increasing from 71% in 65–70-year-olds to 87% in adults aged 80 years and over [7]. There are currently 11 million people aged 65 years and over, representing 17% of the population, which is expected to rise to 25% by 2041 [8].

As the population ages and TBI is anticipated to increase, healthcare organisations face mounting challenges in effectively managing care for older TBI patients. This demographic shift requires more nuanced approaches to patient assessment that go beyond standard injury evaluation to consider the patients overall vulnerability and resilience. Frailty assessment is emerging as a promising framework for addressing this need [9, 10].

Frailty is a consequence of cumulative decline over time which leads to impaired physiological reserve. Following a stressor event, for example in a TBI, frailty may result in increased risk of poor health outcomes. Multiple tools exist for assessing frailty in clinical settings. The comprehensive geriatric assessment is considered a gold standard but is a multidimensional evaluation tool which can help develop an individual integrated care plan [11]. However, it is time-consuming and requires multidisciplinary input, limiting its use in emergency settings. The ‘Fried Frailty phenotype’ faces similar practical constraints [12]. In contrast, the Clinical Frailty Scale (CFS) is widely used in emergency settings due its quick, and simple nature, allowing a rapid screen for level of frailty and has been validated in patients aged 65 years and older [13]. The CFS offers a rapid, pragmatic alternative. It provides a visual and functional assessment based on clinical judgment and patient history, enabling quick evaluation of overall health and functional status. The CFS plays a key role in decision-making for healthcare as a patient’s level of frailty can predict outcomes such as mortality and recovery potential. It also guides decisions on triage, treatments or interventions offered and discharge planning [14].

Despite the growing implementation of frailty assessment tools in various clinical settings, there remains a significant knowledge gap regarding their effectiveness specifically in the context of older adults and the management of TBI. The purpose of this systematic review was to assess the effectiveness of a clinical frailty assessment or scale in older patients following a TBI and its association with patient outcomes.

## Materials and methods

This systematic review was performed in accordance with the Preferred Reporting Items for Systematic Reviews and Meta-Analyses (PRISMA) guidelines. This review was registered with PROSPERO (international registry of systematic reviews) prior to commencement (CRD42022345232).

### Search strategy

The authors performed a search of online databases OVID, EBSCO, Elsevier and Wiley, using the search strategy outlined in Table 1. Only English language studies published between 2005 and 2025 were included, as the Clinical Frailty Scale here has been increasingly adopted into routine practice over the last 20 years.

**Table 1:** Search strategy

Search Strategy on OVID, EBSCO, Elsevier and Wiley database	
1.	MESH terms: “Traumatic Brain Injury” OR “TBI” OR “Head Injury” OR “Brain Injury”
2.	“Adult*”OR “Elderly” OR “Ag*” OR “Older Person” OR “Old”
3.	Search 1 AND 2
4.	“Frail*” OR “Frailty Index” OR “Clinical Frailty Scale”
5.	Search 3 AND 4
6.	“Outcome” OR “Mortality” OR “Complication”
7.	Search 3 AND 5 AND 6
8.	Limit 7 to Human and English Language
9.	Limit 7 to yr = 2005

mp. = title, abstract, heading, keyword, subheading

### Eligibility criteria

Eligible study designs included randomised and non-randomised controlled trials, observational studies, review studies, case-control studies, cross-sectional studies and case series involving a minimum of 10 patients.

Studies inclusion criteria were a minimum of 2/3 of the study population had sustained a TBI (including acute subdural haemorrhage (aSDH), extradural haematoma (EDH), traumatic intraparenchymal haemorrhage (tIPH), traumatic subarachnoid haemorrhage (tSAH) or a combination thereof). The population was predominantly older adults (aged 65 years and older, or a mean participant age), included a validated frailty assessment and report at least one outcome measure (mortality, functional outcome, disability or quality of life).

### Study selection and data extraction

Each abstract was independently screened by two reviewers (EC and NH or CS) with any disagreements resolved through adjudication by a third reviewer (EE). Full text articles of selected abstracts were then reviewed and data extracted independently by two reviewers (EE and NH). And the data extract were discussed and resolved by the third reviewer (EE).

Review papers were only included if they contained original data; otherwise, they were used for backwards referencing only. References from included studies were screened by the same method above and full texts included as appropriate.

All data was extracted into a standardised worksheet including information on study design, number of participants, patient demographics, medical co-morbidities, mechanism of injury, severity of injury, Glasgow Coma Scale (GCS), clinical frailty scale/assessment used, surgical intervention, complications, timepoint of follow-up time, outcomes associated with frailty measures and mortality. The data extraction process was conducted by two independent reviewers (EC and NH), any disagreements were resolved using a third reviewer (EE).

### Quality assessment

Two authors (EC and NH) assessed the quality of studies using the Newcastle-Ottawa Scale (NOS) as no randomised studies were available for inclusion [15]. This tool assesses the risk by considering the selection of study groups, comparability of groups, and outcome. Studies that scored > 6–9 were considered as high quality.

### Statistical analysis

Differences in populations, comparators and outcomes of the included studies did not allow for direct comparison, and therefore meta-analysis was not possible. Instead, the results of these studies were synthesised into narrative and tabular form, with descriptive statistics. Factors previously reported to influence outcome following TBI (admission GCS, patient age and co-morbidities) were comparted across studies. Any significant differences were highlighted within the text and in summary graphs. Data not reported was recorded as missing in the results and excluded from summary statistics. Due to the limited number and wide heterogeneity of studies, only a descriptive summary of any missing outcomes given.

SPSS (IBM Corp, 2023) was used for statistical analysis and data presentation. All data was considered to be normally distributed unless otherwise stated, with means, standard deviations and unpaired T-test for comparing data where appropriate. In non-parametric data, median and interquartile ranges were illustrated.

## Results

### Study characteristics

**Fig. 1:**
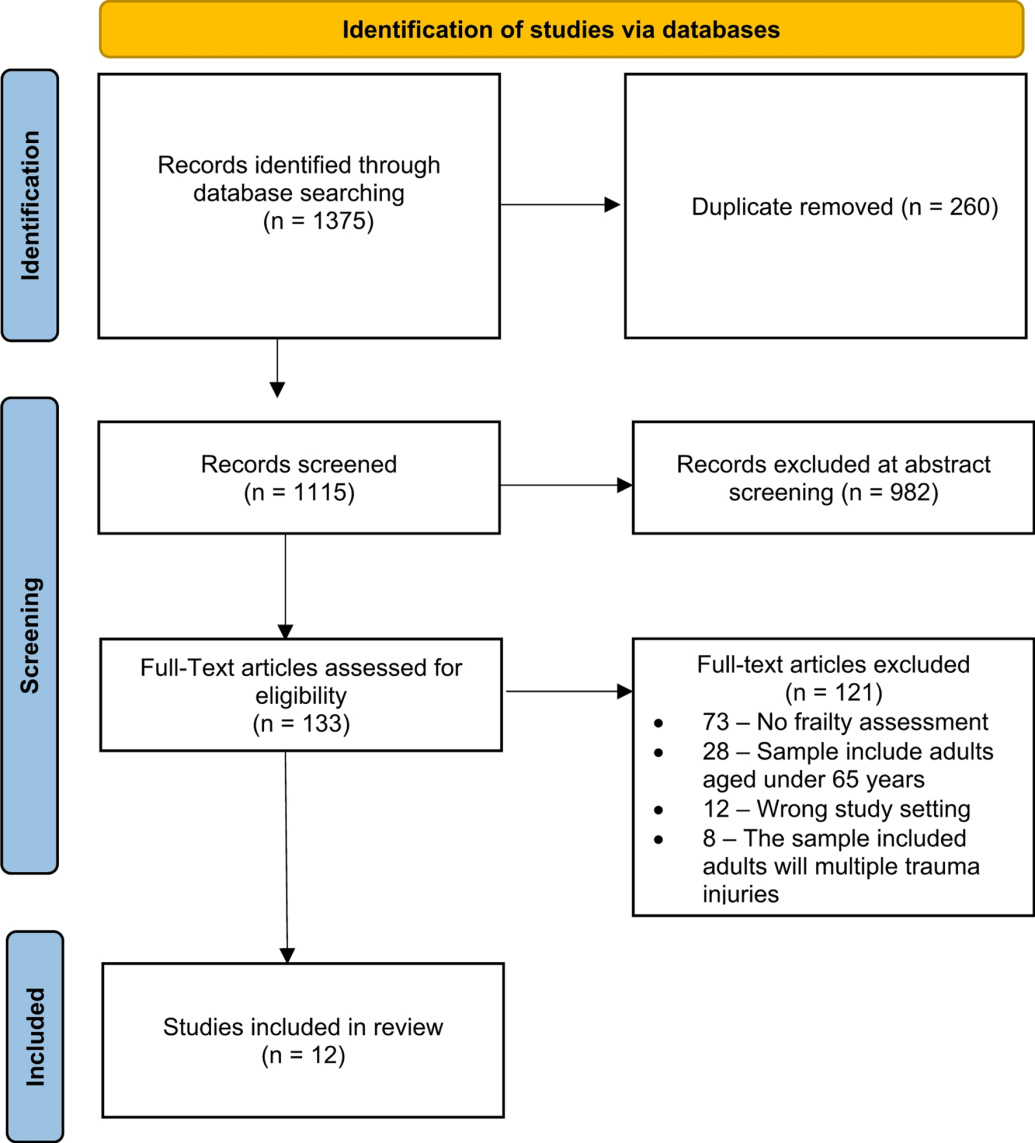
PRISMA 2020 flow diagram. *From*: Page MJ, McKenzie JE, Bossuyt PM, Boutron I, Hoffmann TC, Mulrow CD, et al. The PRISMA 2020 statement: an updated guideline for reporting systematic reviews. BMJ 2021;372:n71. doi: 10.1136/bmj.n71

Following removal of duplicated and ineligible studies, there were 133 full text articles to screen (Fig. 1: article screening) [16]. Twelve articles were included in the final analysis [17–28].

The main characteristics and findings from the included studies are shown in Table 2. The overall number of participants was 464,606 with mean age spanning from 70 to 83 years. There was insufficient data to analyse outcomes by age sub-groups withing this older population. All studies were observational: 10 retrospective and 2 prospective.

**Table 2:** Baseline elements of patient outcome

Study/Year/Country	Design	*N*	Age (range, mean)	Frailty Scale Used	Co-Morbidity definition	Mechanism of Injury	GCS (recorded)	Head Injury Severity	Head Injury Subtype	Timepoint of Follow-Up	Outcome Associated with Frailty	Mortality
Abdulle 2018, The Netherlands [17]	Multi-centre Prospective cohort	161	60–86 (71)	GFI	Comorbidity % (Diabetes, Cardiovascular)– 65%	Fall *N* = 75 Collison *N* = 22 Other Cause *N* = 3	GCS > 13 On admission	ISS *N* = 8.2 (SD.5.9)		3, 6, 12 Months	GOSE	-
Zacchetti 2019, Italy [18]	Single-centre Prospective cohort	60	65–100 (76)	CFS	Comorbidities collected	-	GCS median (IQR) 7(5–10)	-	tSAH *n* = 17(77%)	6 months	GOSE	-
Lee 2020, Australia [19]	Single-centre Retrospective cohort	529	74–85 (80)	mFI-11	Comorbidities collected		Mild TBI GCS 13–15, *n* = 389 Moderate TBI GCS 9–12, *n* = 64 Severe TBI, GCS 3–8, *n* = 75	-	tSDH	30 days 6 Months	GOSE Mortality	30 days
Elsamadicy 2022, USA [20]	Multi-centre Retrospective cohort	2620	18–84 (80)	mFI-5	mFI5 mFI = 1 (*N* = 856) mFI = 2+ (*N* = 672) Number of co-morbidities collected	Assault 9% Fall 51% Pedal cyclist 3% Pedestrian 8% RTA 19% Other 10%	GCS > 3–8 on admission	ISS mFI = 0 *N* = 25 mFI = 1 *N* = 25 mFI2 + *n* = 25		-	LOS	In hospital mortality
Herklots 2022, The Netherlands [21]	Single-centre Retrospective cohort	126	60–94 (70)	CSHA	-	Outdoor Trauma 65 (52%) Indoor Trauma 61 (48%) Outdoor: RTA 53 (82%) Public sites 9 (14%) Work related 2 (3%) Sport 1 (1%) Indoor trauma all but 1 (2%) were falls.	GCS (median) 4.0 (IQR3-7)	ISS (mean) 27 (SD 7)		6 Months	GOSE	6 months
Sastry, Feler 2022, USA [22]	Single-centre Retrospective cohort	143	70–100 (83)	FRAIL	Comorbidities collected	-	GCS on arrival. GCS 13–15 87%	-	-	3 Months	LOS, Discharge location	-
Sastry, Ali 2022, USA [23]	Single-centre Retrospective cohort	100	70–100 (82)	FRAIL	-	-	GCS 14–15 *n* = 89 GCS 9–13 *n* = 6 GCS 3–8 *n* = 4	-	aSDH *n* = 63 (63%) Chronic SDH *n* = 2 (2%) Mix density SDH *n* = 7 (7%) tICH *n* = 20 (20%) tSAH *n* = 33 (33%) EDH *n* = 1 (1%)	Re-admission 30 days.	Discharge Location, Mortality	1 year
Yamamoto 2022, Japan [24]	Single-centre Retrospective cohort	18,065	60–100 (71)	HFRS	-	-	-	-	Diffuse TBI *n* = 1661 Focal TBI *n* = 521 EDH *n* = 637 tSDH *n* = 9560 tSAH *n* = 3618	30 days	Mortality	30 days
Courville 2023, Mexico [25]	Multi-centre Retrospective cohort	381,754	54–81 (70)	MFI-5	-	Blunt injury *N* = 327,246 (96%) Penetrating injury *N* = 2786 (1%) Other cause *N* = 10,064 (3%)	GCS > 13 *N* = 289,772 (85%) GCS 9–12 *N* = 26,464 (8%) GCS < 8 *N* = 23,860 (7%)	ISS Moderate (9–15) *N* = 64,252 (19%) Severe (16–24) *N* = 221,052 (65%) Very severe (> 25) *N* = 54,792 (16%)	aSDH *N* = 226,537 (61%) bSDH *N* = 121,466 (33%) NDSF *N* = 16,885 (5%) Comminuted skull fracture < 2 cm *N* = 3720 (1%)	-	LOS Mortality Discharge location	In hospital mortality
Liu 2024, China [26]	Multi-centre Retrospective cohort	348	70–100	FRAIL	-	RTA *N* = 223 (64%) High Fall *N* = 58 (17%) Stumble N= (12%) Other *N* = 25 (7%)	GCS 13–15 (Mild) *n* = 203 (58%) GCS 9–12 (Moderate) *n* = 58 (16%) GCS 3–8 (Severe) *n* = 88 (25%)	-	Diffuse brain injury *N* = 193 (56%) Focal Brain Injury *N* = 123 (35%) Uncategorised *N* = 32 (9%)	-	Mortality	In hospital mortality
Rafaqat 2024, USA [27]	Single-centre Retrospective cohort	580 (432 TBI)	71–87 (79)	mFI-5	-	Fall *N* = 380 (88%) RTA *N* = 43 (10%) Assault *N* = 3 (1%) Missing data *N* = 6 (1%)	-	ISS Severe (15–24) *N* = 267 (62%) Critical (> 25) *N* = 165 (38%)	SAH *N* = 83 (19%) Parenchymal Contusion *N* = 50 (11%) Parenchymal Haemorrhage *N* = 42 (9%) aSDH *N* = 17 (4%) EDH *N* = 7 (2%)	6–12 months	Re-admission Discharge Location	Functional outcome
Rafieezadeh 2024,USA [28]	Multi-centre Retrospective cohort	60,268	65–100 (76)	mFI-11	-	-	-	ISS Median 18 (IQR 13–25)	EDH *N* = 668 (3%) SAH *N* = 5945 (29%) aSDH *N* = 15,181 (75%) IPH *N* = 1445 (7%)	-	Mortality Discharge Location	In hospital mortality

aSDH, acute subdural haematoma; bSDH, bilateral subdural haematoma; EDH, extradural haematoma; GCS, Glasgow Coma Scale; GOSE, Glasgow Outcome Scale extended; ICH, intracerebral haemorrhage; ISS, Injury Severity Score; LOS, Length of Stay; mFI, modified Frailty Index; NDSF, Nondisplaced skull fracture; RTA, Road traffic accident; TBI, Traumatic brain injury; SAH, subarachnoid haemorrhage; tICH, traumatic intracerebral haemorrhage; tSAH, traumatic subarachnoid haemorrhage; tSDH, traumatic subdural haematoma

The majority of the studies (*n* = 8) presented a low risk of bias, four (*n* = 4) studies presented as moderate risk of bias according to the NOS (Table 3: risk of bias).

**Table 3:** Risk of Bias assessment using Newcastle Ottawa scale (NOS)

Study author, Year	Selection	Comparability	Outcome	Overall risk of bias
Abdulle, 2018 [15]	****	*	***	Low
Zacchetti, 2019 [16]	***		**	Moderate
Lee, 2020 [17]	****	*	***	Low
Elsamadicy,2022 [18]	****	*	***	Low
Herklots, 2022 [19]	****	*	***	Low
Sastry, 2022 [20]	***	*	***	Low
Sastry, 2022 [21]	****	*	***	Low
Yamamoto, 2022 [22]	****	*	*	Low
Courville, 2023 [23]	***	*	**	Moderate
Liu, 2024 [24]	***	*	**	Moderate
Rafaqat 2024 [25]	***	*	***	Low
Rafieezadeh, 2024 [26]	***	*	**	Moderate

### Frailty assessment

Within the 12 studies [17–28], there were seven different frailty measurements used to identify and classify frailty in older patients within an acute clinical setting (Table 4: frailty indices). In each study, frailty was assessed on admission by medical staff. There was only one frailty scale used in each study, with 3 studies utlised the FRAIL scale (Fatigue, Resistance, Ambulation, Illness and weight loss) [29] and 3 studies utlised the 5 item modified frailty index (m-FI) (mFI-5) [30]. These scales can be undertaken at any timepoint without requiring detailed medical review. None of the studies included a frailty phenotype or comprehensive geriatric assessment. The utility of the scales used within the studies must be considered in light of which aspects of frailty they capture, such as physical function, cognition or social factors.

**Table 4:** Frailty indices

Frailty Scale	Assess Co-morbidity	Summary of scale
Clinical Frailty Scale (CFS)	No	9-point scale ranging for very fit (1) to terminally ill (9). Each level based on the individual’s overall physical fitness, functional independence, and vulnerability to health stressors.
Canadian Study of Health and Aging clinical frailty scale (CSHA)	No	7-point scale ranging from very fit (1) to severely frail (7) Each level evaluates an individual’s overall health, physical functioning, and dependence on others for daily activities
FRAIL = Fatigue, Resistance, Ambulation, Illness and weight loss	No	5 Components, each scored as 0 (no) 1 (yes), with a total score ranging 0–5. Scores of 3 or higher indicate frailty, while 1–2 suggest pre-frailty
Groningen Frailty Indicator (GFI)	No	15-item questionnaire with 4 domains: physical, cognitive, social, and psychological functioning. Each item scored 0 (no problem) or 1 (problem) with a total score ranging 0–15. A score of 4 or higher indicates frailty.
Hospital Frailty Risk Score (HFRS)	Yes	Is a calculation using ICD-10 administrative codes that represented pre-existing conditions and then categorised into low (< 5), intermediate (5–15), or high frailty risk (> 15) based on their score
Modified Frailty Index 5-item (mFI-5)	Yes	5-item evaluating diabetes, hypertension, congestive heart failure, chronic obstructive pulmonary disease (COPD), and functional dependence. Each factor is scored as 0 (absent) or 1 (present), with a total score ranging from 0 to 5
Modified Frailty Index 11-item (mFI-11)	Yes	11-item based on the presence of 11 health deficits or comorbidities: diabetes, hypertension, heart failure, COPD, stroke, functional dependence, renal issues, and others. Each item is given a score of 0 (absent) or 1 (present), with the total score ranging from 0 to 11. Higher scores indicate increased frailty.

Frailty scales can include an assessment of co-morbidity (Table 4 frailty indices). Four studies collected preadmission comorbidity/multimorbidity data separately [17–19, 22] (Table 2). The five studies that measured frailty using the mFI-5 or mFI-11 [30, 31] found an association to mortality or unfavorable outcome at 6 months post injury [19, 20, 25, 27, 28]. Increasing frailty on mFI-5/mFI-11 was also associated with increased length of stay (LOS) and a greater likelihood of discharge to a facility or setting other than home [19, 20].

The CFS assessment captures baseline functional status approximately two weeks prior to injury or admission. One study utilising the CFS [13] determined a CFS ≥ 4 served as the optimal threshold to distinguish between frail and non-frail patients when predicting unfavourable outcomes and mortality at 6 months [18]. This finding contrasts widely established frailty definition of CFS ≥ 5, which is conventionally accepted in research for risk stratification purposes [32, 33]. These findings highlight how frailty assessment, regardless of the specific CFS threshold used, serves as fundamental prognostic indicator in acute clinical trajectories for patients with TBI.

### Mechanism of injury

Six studies reported mechanism of injury, with falls (from height and standing) being most common mechanism of injury across all studies [17, 20, 21, 27]. Road traffic collisions represented the second most frequent cause, followed by assault and sports related injuries. The relationship between frailty and injury mechanism varied across studies, with some revealing important associations. One study found TBI caused by collision was twice as prevalent in frail patients compared to non-frail patients (31% v 17%), suggesting that frail individuals may be particularly vulnerable to certain impact mechanisms [17]. WhereasElsamadicy et al., (2022) found increasingly fall-related injuries correlated with higher frailty scores, with 51% of non-frail patients (mFI = 0) and 89% of highly frail patients (mFI = 2+) [20].

When examining the influence of pre-trauma frailty level on injury patterns, one study demonstrated higher frailty scores significantly increased the likelihood of indoor trauma (OR = 2.961, *p* = 0.005) whilst reducing the probability of high-energy trauma mechanisms (OR = 0.300, *p* = 0.002) [21]. This pattern suggests that environmental factors and activity levels differ substantially between frail and non-frail older adults, directly influencing their exposure to different injury mechanisms. Importantly, none of the reviewed studies specifically investigated how different injury mechanisms might affect post-injury changes in frailty status, representing a significant knowledge gap [19].

### Injury severity, GCS and surgical intervention

Injury severity was assessed using the injury severity score (ISS) on admission. Six studies reported ISS [17, 20, 21, 25, 27, 28], nine studies reported on GCS on admission [17–23, 25, 26]. Four studies reported both ISS and GCS scores [17, 20, 21, 25].

GCS was classified using two slightly different grouping approaches were used: either mild (13–15) and moderate (9–12) [19, 26], or mild (14–15) and moderate (9–13) [23]. One study recorded on GCS discharge [23].

Herklots et al., (2022) found that patients with higher frailty had significantly lower ISS scores despite similar GCS values (low frailty: ISS mean 30, SD 11.6; high frailty: ISS 25 (SD 6.4) [21]. In their cohort, participants with high frailty were less likely to experience high-energy trauma (low frailty: 67%, *n* = 33; high frailty: 33%, *n* = 16) and more likely to sustain indoor trauma (low frailty: 37%, *n* = 21; high frailty: 63%, *n* = 36). Frailty remained an independent predictor of mortality after adjustment for ISS.

Two other studies reported no significant differences in ISS across frailty groups [17, 20]. Abdulle et al., (2018) reported an average ISS of 8.2 (SD 5.9) with comparable scores between frail (8.7, SD 6.2) and non-frail (7.9, SD 5.7) [17]. Elsamadicy et al., (2022) found similar ISS values across mFI categories when cohorted into mFI = 0, mFI = 1 and mFI = 2+ (mFI = 0: 25 (IQR 21, 30) v mFI = 1: 25 (IQR 17, 26) v mFI = 2+: 25 (IQR 19, 26) [20].

The relationship between frailty and initial GCS varied across studies. Three studies reported that frail patients were more likely to present with lower GCS scores (≤ 8) on admission, which predicted poorer outcomes [17, 27, 28]. Conversely, two studies using mFI found significantly higher rates of coma (GCS 3–8) in less frail patients (mFI = 0: 49% vs. mFI = 1: 21% vs. mFI ≥ 2: 19%) [19, 20].

Other studies found no association between frailty and GCS.Zacchetti et al., (2019) found no significant difference in the mean GCS in relation to frailty status [18].Herklots et al., (2022) similarly found no significant difference in median GCS values between high and low frailty groups, *p* = 0.455 (High frailty 3.5 (IQR 3–6): Low frailty 4 (IQR 3–7)) [21]. The distribution of frailty across severity categories showed no significant differences: low frailty group mild 91% (*n* = 31), moderate 3% (*n* = 1), severe 6% (*n* = 2); High frailty group: mild (89%) (*n* = 57), moderate 8% (*n* = 5), severe 3% (*n* = 2).

Only one study reviewed surgical intervention following a traumatic acute subdural haematoma but it did not explore any associations with frailty, mortality or outcomes [19]. No other studies assessed surgical interventions.

### Timepoint to follow-up

Eight studies followed patients up over set time points [17–19, 21–24, 27]. One study followed patients up at multiple time points − 2 weeks, 2-, 6- and 12-months following injury [17]. Further additional questionnaires were sent between 1 and 3 years post injury regarding quality of life, functional outcome, but the mean follow-up time was only 6 months.

Four studies followed patients up at 6 months post injury [18, 19, 21, 27], with three utilising the extended Glasgow outcome scale (eGOS) [34] as their secondary study outcome at 6 months [18, 19, 21].

Other studies included short-term outcomes, such as 30-day readmission or mortality [22, 24], and one reported follow-up data at 3 months, although only 52% attended their appointment [23]. These varying follow-up periods highlight inconsistency in outcome reporting, though mortality and functional limitations remain frequent endpoints.

### Mortality and frailty

Four studies examined the relationship between frailty and mortality [19, 24, 26, 28].Lee et al., (2020) found patients with increasing frailty on admission to hospital was associated with increasing mortality at 30 days when cohorted into mild and moderate TBI (Mild OR 1.39, 95% CI 1.09–1.76, *p* = 0.008; Moderate OR 1.71, 95% CI 1.02–2.87, *p* = 0.042) [19]. The mFI was found to be a predictor of increased mortality at 30 days when comparing mFI 0/11 (18% mortality) to mFI ≥ 5/11 (30% mortality). In the severe TBI group (*n* = 10) patients with an mFI ≥ 3/11, no survivors were observed.

Rafieezadeh et al., (2024) found that while mFI as a continuous variable was not a significant predictor of mortality (*p* = 0.058), categorised frailty status did not predict mortality (OR = 1.786, *p* = 0.026) [28]. Co-occurrence of ≥ 2 types of intracranial haemorrhage also significantly increased mortality risk (OR = 2.251).

Liu et al., (2024) found that high frailty, as assessed by the FRAIL scale, was an independent predictor of in-house mortality (*P* < 0.001, OR = 2.012, 95% CI: 1.788–2.412) [26].Yamamoto et al., (2022) observed a twofold increase in 30-day mortality among high frail patients (Low-frailty 7%, High-frailty 16%; *p* < 0.001) [24]. These findings position frailty as a reliable prognostic indicator, independent of GCS or ISS.

### Functional outcome and frailty

Four studies utilised the eGOS to evaluate the relationship between frailty and functional outcomes [17–19, 21]. The eGOS is a validated 8-point scale for assessing neurological outcomes in patients with brain injuries [34]. All four studies found increasing frailty to be a significant predictor of unfavourable outcomes at 6–12 months post injury.Abdulle et al., (2018) dichotomised eGOS into favourable (eGOS 8) and unfavourable outcomes (eGOS 1–7), finding that increasing frailty was associated with unfavourable outcome at 12 months post injury (OR, 1.87; 95% CI, 1.51–2.31; *p* < 0.001) [17]. Similarly, Herklots et al., (2022) dichotomised differently - favourable (eGOS ≥ 5) and unfavourable (eGOS ≤ 4) and reported comparable findings 6 months post injury (OR 4.35; *p* = 0.03) [17, 21].

Lee et al., (2020) demonstrated a clear association between increasing frailty and poorer functional outcomes. The proportion of participants experiencing unfavourable outcomes at six months post injury, rose markedly from 41 to 98% in those with as mFI increasing from 0 to ≥ 5/11 [19]. Rafaqat et al., (2024) reported that frail TBI patients experienced significantly greater functional limitations in activities of daily living (ADL) at 6–12 months post-injury (68% vs. 49%) [27]. Functional limitations were defined as the inability to independently perform one or more ADL, specifically, walking, stair navigation, personal hygiene, meal preparation and toileting.

### Hospital length of stay and discharge location

Three studies assessed hospital length of stay (LOS) in relation to frailty [20, 22, 25].Elsamadicy et al., (2022) found that patients with increased frailty (mFI = 2+) was associated with a longer LOS (OR 1.4, 95% CI, 1.03–1.9, *p* = 0.03) and older age contributed to longer stays [20].Courville et al., (2023) found that sustained LOS increases with increasing frailty, patients had a median LOS of 5 days (IQR: 2.2, 9.7) with a sustained increased for frail (5.2 days (IQR 1.7, 10.1) and very frail (5.3 days (IQR: 0.5, 11.0) patients [25]. Frail patients were more likely to be discharged to higher level of care facility (community hospital, nursing or care home) (54%).

One study found no association with frailty and LOS but did show a significant difference in unfavourable discharge destination (discharge other than home) between frailty categories using univariate analysis (non-frail 19%, prefrail 58%, frail 66%,*p* = 0.001) [22]. Another study found a high frailty was associated with higher rates of non-home discharge (54% v 26%, *p* = 0.007) [23].

Rafieezadeh et al., 2024 did not specifically analyse or report on length of hospital stay in relation to frailty [28]. However, their secondary outcome investigating an unfavourable outcome which was defined discharge location other than home. They found that higher mFI score significantly predicted unfavourable discharge outcome (OR = 4.841, *p* < 0.001) in 50% of patients.Rafaqat et al., (2024) also collected data on discharge disposition (home, home with services, rehabilitation, nursing home, other), but they found no significant difference between frail and non-frail patients in their discharge locations (*p* = 0.78) [27].

## Discussion

This review assessed the effectiveness of clinical frailty assessment tools in predicting outcomes in older TBI patients (≥ 65 years). While we intended to evaluate their effectiveness in predicting outcome and quality of life, the paucity of data in this field makes this impossible, and only allowed analysis of correlations with functional outcome and mortality. Despite frailty being well investigated in many clinical settings, it remains understudied within neurosurgical literature and this TBI patient group [35–37]. Studies in other specialties have demonstrated that frailty assessments can be effective in predicting outcomes such as hospital admission or mortality, particularly in combination with other measures [9, 10, 38]. As the world’s population ages, priortising frailty research becomes essential, especially as previous studies suggest frailty may be a better metric for predicting outcomes than chronological age. The absence of validated quality of life assessments like EQ-5D in this population is an area of particular concern and needs urgently addressing in future studies.

Our findings demonstrate that various frailty measures effectively predict mortality, hospital length of stay, discharge disposition and functional recovery, with enhanced prognostic value when combined with traditional neurosurgical metrics like GCS and ISS. However, a major limitation identified across studies is the absence of validated quality of life assessments. Without this data, it is difficult to fully understand the broader, patient-centred implications of frailty following TBI. Additionally, significant variation in assessment tools presents both challenges and opportunities. Standardisation in frailty assessment is crucial, as inconsistencies may introduce bias and hinder comparability of outcomes across studies and clinical settings.

This review highlights significant variation in frailty assessment tools utilised in TBI patients. Such heterogeneity presents both a challenge and an opportunity for more nuanced patient evaluation. The FRAIL scale demonstrated effectiveness in predicting patient outcomes [22, 23], although its primary application in outpatient settings limits its direct relevance to acute TBI care. Similarly, the mFI 5 and mFI 11 demonstrate promise in trauma and surgical environments, particularly when combined with GCS scores for risk stratification [19, 20]. While not a direct measure of functional status, the mFI’s correlation with 30-day and 1 year mortality provides valuable insights into patient vulnerability. Notably, when combined with GCS scores mild (GCS 13–15) and moderate (GCS 9–12), the mFI emerged as a critical tool for risk stratification [19].

The CFS, widely used within UK healthcare, offers validated predictive ability across multiple health settings [39–41]. Our analysis showed that frailty is a significant predictor or multiple outcomes in older TBI patients, which include mortality, length of hospital stay, discharge disposition and functional recovery. However, a significant limitation emerged, whilst the CFS is valuable, it cannot standalone in assessing older patients with a TBI as it fails to account for global neurological function specific to TBI. This underscores the need for a comprehensive approach that combines frailty assessment with tools like GCS to predict outcomes in neurosurgical patients.

The timing of frailty assessments represents another critical area with insufficient data. Studies rarely specified when in the patient journey assessments occurred (emergency department, acute medical ward, or later during inpatient stay) or who conducted them. Some measures like the mFI rely entirely on pre-existing co-morbidities, potentially calculated retrospectively from medical notes, rather than through clinical judgement at injury time. Inter-rater variability further complicates assessment reliability, with studies showing moderate to good reliability among emergency department staff (ICC 0.78) [33] but only fair agreement between intensivists and geriatricians (Cohen’s kappa 0.32) [42]. This highlights the influence of assessor background on frailty scoring and indicates the need for standardised training and protocols to enhance the consistency and reliability of frailty assessments across different clinical settings and professional backgrounds.

Frail patients demonstrated notably reduced functional recovery following TBI, showing slower recovery progress, increased dependency on mobility aids and significant limitations in daily activities [43]. One study reported that 46% of frail patients experienced some form of disability with decreased quality of life [17]. These findings extend beyond TBI, resonating with broader observations of frailty’s impact on patient outcomes with very frail patients unlikely to recover back to baseline.

A critical gap in the existing research is the lack of comprehensive exploration of confounding factors. Our review identified several key challenges with methodological limitations such as; inconsistent frailty assessment tools, limited data on pre-existing comorbidities and challenges in distinguishing TBI-related decline from frailty-associated decline. The confounding factors were multiple comorbidities, cognitive impairment and variability in patient baseline health status. These complexities underscore the need for more sophisticated, nuanced research designs that can effectively isolate and understand the multifaceted nature of frailty in older patients following a TBI.

This systematic review highlights the critical importance of frailty assessment in older TBI patients. By incorporating frailty evaluation into prognostic models, clinicians may be able to enhance outcome prediction and develop more individualised care strategies [44]. Tailoring interventions and resource allocation based on frailty status has the potential to, improve patient outcomes and optimise healthcare interventions for this vulnerable population. Future research should focus on, standardising frailty assessment tools, developing integrated assessment protocols and exploring long-term functional and quality of life in older adults following a TBI.

## Conclusion

This systematic review demonstrated that clinical frailty assessments are effective in predicting key patient outcomes in older adults following a TBI. Our analysis revealed that various frailty assessment tools can potentially predict adverse outcomes, including: increased mortality rates, extended hospital stay, decreased functional recovery and greater dependence on post-discharge support services.

The integration of validated frailty scales into routine admission protocols represents a promising approach to early risk identification and personalised patient management. However, significant research gaps remain. Future prospective studies should focus on: validating specific frailty scales across diverse demographic groups, longitudinal tracking of long-term patient functional and quality of life outcomes and the development of standardised, neurologically relevant assessment protocols.

A deeper understanding of the interplay between frailty and TBI outcomes in older patients is crucial for advancing geriatric neurocritical care and optimising patient-centred approaches to treatment and rehabilitation.

## Data Availability

No datasets were generated or analysed during the current study.
